# What’s new and notable in bacterial spore killing!

**DOI:** 10.1007/s11274-021-03108-0

**Published:** 2021-08-05

**Authors:** Peter Setlow, Graham Christie

**Affiliations:** 1 Department of Molecular Biology and Biophysics, UConn Health, Farmington, CT 06030-305 USA; 2 Department of Chemical Engineering and Biotechnology, University of Cambridge, Cambridge, CB3 OAS UK

**Keywords:** Bacillus, Spores, Spore killing, Spore resistance

## Abstract

Spores of many species of the orders Bacillales and Clostridiales can be vectors for food spoilage, human diseases and intoxications, and biological warfare. Many agents are used for spore killing, including moist heat in an autoclave, dry heat at elevated temperatures, UV radiation at 254 and more recently 222 and 400 nm, ionizing radiation of various types, high hydrostatic pressures and a host of chemical decontaminants. An alternative strategy is to trigger spore germination, as germinated spores are much easier to kill than the highly resistant dormant spores—the so called “germinate to eradicate” strategy. Factors important to consider in choosing methods for spore killing include the: (1) cost; (2) killing efficacy and kinetics; (3) ability to decontaminate large areas in buildings or outside; and (4) compatibility of killing regimens with the: (i) presence of people; (ii) food quality; (iii) presence of significant amounts of organic matter; and (iv) minimal damage to equipment in the decontamination zone. This review will summarize research on spore killing and point out some common flaws which can make results from spore killing research questionable.

## Introduction

### The problem

Spores of many species of the orders Bacillales and Clostridiales are dormant and extremely resistant to all manner of killing regimens and can survive for long periods in the environment and on a variety of surfaces (Leggett et al. 2012; Setlow and Johnson 2012). As a consequence, these species can readily contaminate foods or surfaces with which humans come in contact. Accordingly, spores of some species act as vectors for a number of human diseases and intoxications, including severe diarrhea caused by *Clostridioides difficile*, intoxication caused by *Clostridium botulinum* or *Clostridium perfringens*, and food poisoning caused by *Bacillus cereus*. Spores of many species are also important vectors for spoilage of a number of types of foods, including juices, bread and dairy products. Finally, spores of one species, *Bacillus anthracis*, are a potential biological weapon, and was used in this manner in the United States in 2001. Given the potential deleterious effects of spores, there has been and still is much interest in methods to kill spores (Cho and Chung 2020; Reineke and Mathys 2020; Setlow 2006, 2013).

Concerns about spores are exacerbated due to their extreme resistance properties, including to UV and γ-radiation, wet and dry heat, desiccation, high pressures, plasma, and a host of chemical agents including acids, bases, alkylating agents and oxidizing agents (Cho and Chung 2020; Reineke and Mathys 2020; Setlow 2006). As a consequence of their extreme resistance, spores are difficult to kill, but, thankfully, they can be killed. Indeed, a large amount of research has been, and continues to be carried out on ways to kill spores and to determine the mechanism of such spore killing. The overarching purposes of this review are to discuss: (1) why spores are so resistant to killing; (2) how spores are killed by various agents; and (3) various pitfalls that bedevil research on spore killing.

### Spore formation

Spores are formed via the process of sporulation which is most often triggered by starvation. Early in their sporulation, cells of almost all species of spore-forming species in Bacillales and Clostridiales undergo a special asymmetric sporulation division giving rise to a larger mother cell and a smaller forespore or prespore compartment. The latter compartment is engulfed by the mother cell and subsequently the two compartments exhibit different transcriptional and translational programs. Ultimately, when spore development has progressed far enough, the mother cell lyses to release the now dormant spore. However, the released spore still undergoes some changes that affect its properties, most notably significant amounts of coat protein cross-linking, some of which almost certainly affect spore resistance properties (Abhyankar et al. 2015; Sanchez-Salas et al. 2011). It is also important to appreciate that variables in sporulation conditions including using liquid or solid media, nutrient and divalent cation levels, and pH in the sporulation medium and temperature, can all affect the resistance properties of the spores produced (Bressuire-Isoard et al. 2018; Melly et al. 2002; Rose et al. 2007).

### Spore morphology and behavior

**Fig. 1:**
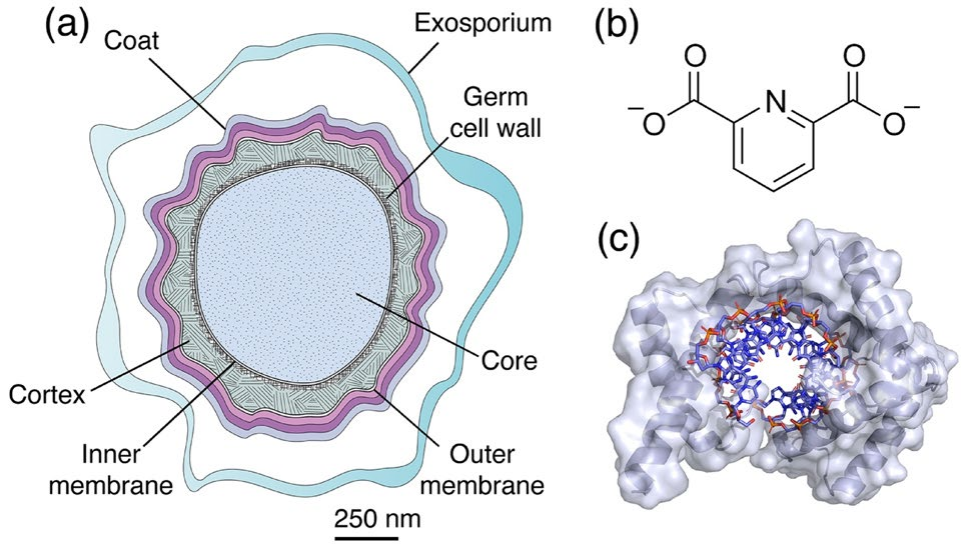
**a** Schematic of a spore showing the main structural features that are present in spores of most species of Bacillales and Clostridiales (some lack an exosporium). **b** Pyridine-2,6 -dicarboxylic acid (DPA). **c** Crystal structure of SASP proteins (grey) bound to A-type DNA (blue and red)

Spores of Firmicutes have a very different structure than growing cells, with many layers and components that are unique to spores (Fig. 1a), and many of these unique layers/components are important in spore resistance properties. The outermost exosporium layer is not found in spores of all species and any specific role in spore resistance has not been definitively established. The next layer, the coat, is found in all species, is composed of proteins unique to spores and often has several different layers. The coat is important in spore resistance to reactive chemicals such as chlorine dioxide, hypochlorite and ozone (Sanchez-Salas et al. 2011; Young and Setlow 2003, 2004b) and extremely important in spore resistance to being eaten and digested by at least several types of bacteriovores (Klobutcher et al. 2006; Laaberki and Dworkin 2008). Under the coat is the outer membrane (OM), which may not be a complete membrane in spores. The OM is readily removed by decoating regimens which decrease spore resistance to wet heat, hydrogen peroxide, DNA damaging chemicals and at least some radiation (Clair et al. 2020; Cortezzo et al. 2004; Cortezzo and Setlow 2005). Decoating does, however, greatly decrease resistance to oxidizing agents that most likely damage the inner membrane (IM) (Cortezzo et al. 2004). Next is a large peptidoglycan (PG) layer, the cortex, whose structure is different to that of growing cell PG. The cortex appears to play an important role in reducing the water content in the spore core, which is important in spore dormancy and resistance to wet heat (Rao et al. 2016). Under the cortex is the germ cell wall with a PG structure identical to that in the growing cell wall. Then comes the IM, which is composed of phospholipids and fatty acids similar to those found in the growing cell plasma membrane. However, lipid probes in the spore IM are largely immobile (Cowan et al. 2004), and the permeability of the IM is very low, even to water (Knudsen et al. 2016). The low IM permeability appears to play an important role in spore resistance to some reactive chemicals, in particular chemicals that can damage DNA (Setlow 2006; Tennen et al. 2000). Some of the IM is also present as vesicles that extrude into the central spore core (Laue et al. 2018) and which presumably are incorprated into the expanding membrane that is observed during spore germination. The final layer is the core, where spore DNA, RNA and most spore enzymes are located. A major feature of the spore core is its very low water content, even when spores are suspended in water, and this can be as low as ~ 25% of wet weight. The core water content is likely important in spore dormancy, and of most importance in spore wet heat resistance, as there is a good inverse relationship between spores’ core water content and wet heat resistance (Beaman and Gerhardt 1986; Coleman et al. 2007, 2010). As noted above, the cortex PG appears to play some role in the core’s low water content. Another core component also plays a major role in spore core water content, and this is a 1:1 chelate of Ca^2+^ and dipicolinic acid (CaDPA) which makes up ~ 25% of the dry weight of the core (Fig. 1b) (Setlow et al. 2006). DPA is made in the mother cell compartment of the sporulating cell and taken up almost certainly as CaDPA late in sporulation, displacing core water. This molecule is present at far above its solubility concentration and may be in the core in a crystalline form. Another unique spore component is a group of novel small, acid-soluble spore proteins (SASP) of the α/β-type (Lee et al. 2008). These proteins are found in spores of all species examined and appear to be a part of the fundamental protein complement of spores. The a/b-type SASP are DNA binding proteins (Fig. 1c), and saturate spore DNA and protect it from many damaging agents (Moeller et al. 2008). It is also notable that the pH in the spore core is ~ 6.5, > 1 pH unit lower than in growing cells, although whether the slightly acidified spore core environment contributes significantly to spore dormancy has not been established.

### Spore germination

While spores are dormant and resistant and can survive in this state for many 100 s of years, given the proper environment spores can return to life in the process of germination within minutes (Christie and Setlow 2020). These “proper” environments that can trigger germination contain small molecules termed germinants, which are often specific small molecules, some of which can be nutrients, that signal to spores’ germinant receptors that the spore’s environment is now conducive to growth of the organisms. Within minutes of exposure to germinants, spores begin the germination process such that the CaDPA in the core is released via an IM CaDPA channel, and the cortex PG is hydrolyzed by spore-specific cortex-lytic enzymes (CLEs). These changes result in water uptake by and expansion of the spore core such that core enzymes become active and metabolism and macromolecular biosynthesis resume. However, metabolism and macromolecular synthesis are most likely not required for complete germination (Setlow and Christie 2020; Swarge et al. 2020a, b). The order of CaDPA release first and then cortex PG hydrolysis is seen in germination of spores of Bacillales and some Clostridiales species, while in other Clostridiales, cortex PG hydrolysis precedes CaDPA release (Setlow et al. 2017). However, completion of both processes in either order gives a fully germinated spore, which is then converted to a growing cell via the process of outgrowth.

## Spore killing

There are three important questions to be dealt with when discussing spore killing, and these are: (1) what are the factors responsible for spores’ resistance to killing by various agents; (2) how are the spores killed by various agents; and (3) are supposedly killed spores truly dead or are they in suspended animation and only awaiting Prince Charming’s kiss to revive them?

### Factors involved in spore resistance to killing by various agents

As was mentioned briefly above, different spore attributes are involved in resistance to killing by different agents. The coat protects against hydrolytic enzymes that degrade PG (Mundra et al. 2014), including hydrolytic enzymes in bacteriovores (Klobutcher et al. 2006), and also acts as reactive armor to detoxify some harmful chemicals e.g., detoxifying enzymes such as superoxide dismutase and catalase are often present in the spore coat. However, the apparent molecular sieving properties of the coat do not extend to potentially damaging small molecules, which if not enzymatically detoxified, can diffuse deeper into the spore. Since neither the OM or cortex appear to present significant permeability barriers, it is the function of the IM to impede access of many harmful chemicals to the spore core. These include DNA damaging agents and some chemicals that can damage proteins (Cortezzo et al. 2004; Mokashi et al. 2020). The low water content in the core protects core enzymes against wet heat damage, and both CaDPA accumulation and cortex action contribute to the core’s low water content (Coleman et al. 2007, 2010; Coleman and Setlow 2009; Rao et al. 2016). The α/β-type SASP also protect DNA against wet heat damage, and CaDPA may assist in protecting DNA from some damage by immobilizing it (Setlow et al. 2006), although generally not against radiation induced damage. Notably, recent work has found an additional factor capable of protecting spores against wet heat, which is genes expressed from a transposon found in spores of some *Bacillus* species making extremely wet heat-resistant spores (Krawczyk et al. 2016, 2017; Luo et al. 2021; Tu et al. 2021). The same transposon is also associated with enhanced resistance against high pressure (Li et al. 2019). However, the mechanisms of this protection against wet heat and high pressure are not yet clear. The α/β-type SASP are most important in protecting spore DNA against lethal damage by UV and γ-radiation, and also play a role in spore protection against wet and dry heat as well as some genotoxic chemicals, as has been shown using spores, termed α^−^β^−^, that lack the majority of these proteins (Moeller et al. 2008; Schottroff et al. 2019; Taylor et al. 2020). Spores also contain many DNA repair proteins, including one active only on a UV photoproduct specific to spores, termed the spore photoproduct (SP) (Moeller et al. 2008). These DNA repair enzymes do not work in the dormant spore, but only rather soon after spore metabolism and macromolecular synthesis resume after initiation of germination.

### How are spores killed by various agents?

Spores have a number of possible targets, damage to which could result in spore death including: (i) DNA in the core; (ii) the IM, damage to which can alter the permeability of this membrane and its structural stability; and (iii) proteins in the core that will be essential for reactions essential for spore resumption of growth, such as in central metabolism. Indeed, damage to all of these possible targets have been identified as a means to kill spores (Table 1).

**Table 1:** How to kill a spore

Category	Sub-category	Specific agent(s)	Key spore component(s) damaged	Key spore resistance feature	References
Physical processes	Heat	Dry heat	DNA	SASPs	He et al. (2018), Johansson et al. (2011)
		Wet heat	Essential core protein(s)	Low water content; SASPs; Transposon associated genes	Coleman et al. (2007, 2010), Coleman and Setlow (2009), Huesca-Espitia et al. (2016), Krawczyk et al. (2017), Rao et al. (2016)
		Ohmic			Schottroff et al. (2019)
		Thermosonication	Inner membrane		Fan et al. (2019)
		High temperature gas	Core proteins		Setlow et al. (2014)
	Pressure	High hydrostatic pressure	Inner membrane proteins		Doona et al. (2016), Modugno et al. (2019), Morimatsu et al. (2019)
		Moderate pressure plus heat			Morimatsu et al. (2019)
		High pressure CO_2_ plus heat	Inner membrane		Rao et al. (2019)
	Radiation	UV-C (222 nm, 254 nm, pulsed light)	DNA	SASPs, DNA repair enzymes, Coat	Clair et al. (2020), Narita et al. (2020), Taylor et al. (2020)
		Blue light	DNA	Coat, pigmentation, SASPS, DNA repair enzymes	Djouiai et al. (2018)
		Ionizing	DNA	SASPs, DNA repair enzymes	Moeller et al. (2008)
		Electron beam	DNA	DNA repair enzymes	Zhang et al. (2020)
Chemical processes	Strong acids	HCl	Inner membrane	Low permeability	Setlow et al. (2002)
	Strong bases	NaOH	CLE damage	Low permeability	Setlow et al. (2002)
	Oxidising agents	Hypochlorite, chlorine dioxide, ozone	Inner membrane	Coat	Cortezzo et al. (2004), Setlow et al. (2013), Stier and Kulozik (2020), Young and Setlow (2003, 2004a)
		Hydrogen peroxide	Core protein(s)	SASPs, Coat	Moeller et al. (2011), Setlow and Setlow (1993), Setlow et al. (2013), Stier and Kulozik (2020)
	Surfactants, lipids, natural oils	Dodecylamine; Ceragenin CSA-13; CTAB	Inner membrane		Alqadeeri et al. (2019), Cho et al. (2015), DeMarco et al. (2021), Dong et al. (2019), Mokashi et al. (2020), Piktel et al. (2017)
	Alcohols and aldehydes	Ethanol; gluteraldehyde	Inner membrane	Coat (for glutaraldehyde)	Player et al. (2020), Setlow et al. (2002), Tennen et al. (2000)
	Gases, vapours, plasma	Supercritical CO_2_ + peracetic acid; Iodine vapour; Hydrogen peroxide vapour;	Inner membrane [HPV to be determined]		Jiang et al. (2018), Mickelse et al. (2019), Rutala et al. (2020), Setlow et al. (2016)
Biological processes	Enzymes	Proteases	Spore coat	Coat	Mundra et al. (2014)
		Phage lysins	Cortex	Coat	Fu et al. (2020)
	Germinate to eradicate	Amino acids, taurocholate; can be coupled with antibiotics, heat and or surfactants	Germinant receptors	Variable response to germinants	Budi et al. (2021), Buhr et al. (2020), Cho et al. (2015), Nerandzic and Donskey (2010, 2013)

#### Damage to spore DNA

UV or γ-radiation can damage spore DNA generating lethal mutations resulting in spore death (Fayolle et al. 2020; Khodadad et al. 2017; Kim et al. 2020; Moeller et al. 2011, 2014; Narita et al. 2020; Nerandzic et al. 2015; Taylor et al. 2020). UV damage by radiation at 254 or 222 nm also generates a novel photoproduct in spore DNA, a thyminyl-thymine adduct between adjacent thymine residues termed SP which is a potentially lethal photoproduct (Setlow 2006). Exposure of spores to intense blue light at a wavelength of 400 nm also results in SP formation and spore inactivation (Djouiai et al. 2018). However, SP can be repaired after germination is complete by multiple pathways including via spore-associated SP lyase activity, and defects in SP repair due to lack of SP lyase or other proteins of SP repair increase spore killing by UV (Djouiai et al. 2018; Moeller et al. 2008; Setlow 2006, 2007). Notably SP is formed in spore DNA because of the novel structure adopted by DNA saturated with a/β-type SASP (Lee et al. 2008; Moeller et al. 2008). γ-Radiation and a range of other ionizing and/or high energy beams also kill spores by DNA damage, some of which can again be repaired by germinated spores (Granger et al. 2011; Moeller et al. 2014; Nerandzic et al. 2015; Setlow 2006; Zhang et al. 2020). This damage includes strand cleavage and oxidation of DNA bases, predominantly guanine residues.

Other agents that kill spores by DNA damage include dry heat (He et al. 2018; Johansson et al. 2011; Setlow et al. 2014) and genotoxic chemicals such as formaldehyde and nitrous acid (Melly et al. 2002; Player et al. 2020; Setlow et al. 2002; Setlow 2006, 2013; Tennen et al. 2000). The α/β-type SASP are most important in resistance to these agents, as is the IM permeability barrier. Notably, hydrogen peroxide, which can certainly cause DNA damage in growing cells, does not kill spores by DNA damage because of DNA protection by α/β-type SASP, and this is also true for wet heat (Cortezzo et al. 2004; Setlow et al. 2013; Setlow 2007, 2013).

#### Damage to the inner membrane

A variety of agents have been implicated in causing IM damage, including oxidizing agents such as hypochlorite and chlorine dioxide which are used in applied settings (Cortezzo et al. 2004; Setlow et al. 2016, 2013). The evidence that these agents cause IM damage is that (i) damage to other spore components has not been detected, and (ii) these agents do not even kill α^−^β^−^ spores via DNA damage, although these agents can cause DNA damage in growing cells. Presumably this is because the spore IM is largely impermeable to these agents. Other evidence on how these types of agents kill spores is also consistent with IM damage, in particular that with survivors of spores killed by these agents: (i) CaDPA is released with lower temperature heat treatments than untreated spores and that the germinated-killed spores rapidly lyse; (ii) survivors are greatly sensitized to subsequent wet heat treatment; (iii) survivors become very sensitive to high NaCl in recovery on plates, which again is a characteristic of membrane damage, although certainly not unique to this; (iv) survivors often become sensitive to heat, desiccation and high osmotic strength recovery media; (v) such treated spores exhibit an increased rate of spore germination with dodecylamine which acts on the IM (see below); and (vi) and most telling, dormant survivors exhibit remarkable increases in IM permeability (Cortezzo et al. 2004).

**Fig. 2:**
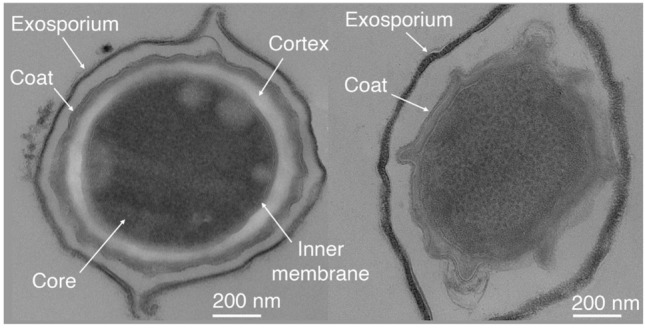
Thin section transmission electron micrograph of a dormant *Bacillus megaterium* spore (left), with major structural features highlighted. The spore on the right has been exposed to the cationic detergent dodecylamine, which acts on the inner membrane to stimulate a series of germination-like responses. Dodecylamine is sporicidal at sufficiently high concentration

Another IM damaging agent that kills spores has been identified, and is the aforementioned alkylamine dodecylamine (Mokashi et al. 2020). This agent can trigger spore germination by activating the SpoVA channel for CaDPA release, but spore killing by dodecylamine does not involve or require spore germination (Fig. 2). Instead, dodecylamine kills spores by destroying the IM permeability barrier such that all core small molecules are released (Mokashi et al. 2020). Presumably when these dodecylamine-treated spores germinate they are unable to maintain the proton motive force (PMF) that is needed for bacterial viability (note that dormant spores do not have a PMF across the IM). Notably, dodecylamine kills spores of all species that have been tested, including *Bacillus* species and *Clostridioides difficile* (DeMarco et al. 2021).

#### Damage to essential core proteins

Since proteins in the spore core will be essential for metabolism in the germinated/outgrowing spore, if one or more are significantly damaged this might compromise the ability of the germinated spore to survive (Coleman et al. 2007; Setlow et al. 2014). Indeed, it has been shown that wet heat-killed spores of several species can germinate, but the germinated spores make little if any ATP (Coleman et al. 2010). This suggests that some enzyme essential in metabolism is inactivated by wet heat. Notably, wet heat-killed spores that retain their CaDPA can germinate and retain their IM permeability barrier, but have accumulated some protein denaturation as assessed by Raman spectroscopy of individual spores (Setlow et al. 2013). Hydrogen peroxide is another commonly used sporicide. This agent can kill spores of strains that lack most α/β-type SASP by DNA damage (Setlow and Setlow 1993). Thus, H_2_O_2_ can enter the spore core. However, H_2_O_2_ does not kill wild-type spores by DNA damage, but most likely by inactivation of some key core protein(s) (Cortezzo et al. 2004; Moeller et al. 2011; Setlow et al. 2013).

### Are ‘killed’ spores truly dead?

It also needs to be appreciated that treatment with many agents, including wet heat, dry heat, radiation and many chemicals can cause damage to spores that may be conditionally lethal, but only under some recovery conditions and not others. Thus, spores with DNA damage leading to auxotrophy may not die in rich media. This can also be true for some percentage of spores in populations treated with wet heat or oxidizing agents. While some spores appear truly dead, others are only damaged and if recovered, generally on a very rich medium that often includes glucose, can grow and the damage is then not permanent (Coleman and Setlow 2009; Rao et al. 2016; Russell and Loosemore 1964). Damaged spores can also be more sensitive to elevated temperatures or salt concentrations in recovery medium, so it is generally essential to test such variables to be sure treated spores are truly dead (Camilleri et al. 2020).

#### Destruction of spores’ ability to germinate

Clearly, a spore incapable of germinating will present as a dead spore. However, if the spore is otherwise normal, the spore might be recovered by artificial germinants. Thus, when any putative killing method is used for spore eradication, it is essential ensure that the apparently killed spores are truly dead (Setlow 2019). Notably, spores killed by many of the agents listed above can often still germinate, albeit sometimes slower than untreated spores. This is true for spores killed by radiation, dry and wet heat and many chemicals (Mtimet et al. 2017; Rao et al. 2016; Russell and Loosemore 1964; Setlow et al. 2013). Agents which target proteins involved in germination include wet heat and some chemicals. The most dramatic of the latter is NaOH, as spores treated with strong base can appear dead on nutrient plates, but are recovered well on plates with low levels of lysozyme (Setlow et al. 2002). The latter observation, as well as that NaOH-treated spores release CaDPA following germinant addition but never complete germination, suggests that strong alkali inactivates all spore CLEs which are outside the spore core, but the core components can be undamaged. Lysozyme at low levels then takes the place of CLEs in germinating the dormant spores which presumably have had sufficient coat proteins removed by the NaOH to allow lysozyme to access the cortex PG (Atrih and Foster 2001; Rao et al. 2016; Setlow et al. 2002).

## Potential problems encountered in spore killing research

### Using impure spores

A common problem in spore killing research is the use of spore preparations that are not especially pure, with large amounts of debris remaining from lysed mother cells or cells which did not sporulate (Green et al. 2020; Schlievert et al. 2018). This debris can include nucleic acids, proteins and cell wall fragments, and this can interfere with spore killing by radiation, heat and chemicals, such that reproducibility of killing regimens between labs can be poor and the design of regimens for spore killing potentially more difficult than it ought to be. Consequently, it is imperative that laboratory studies on efficacy of spore killing and its mechanism be carried out with spores that are well purified and with the maximum amounts of debris removed. Procedures for this involving density gradient centrifugation, lysozyme treatment, and adequate cycles of sedimentation and resuspension in deionized water, have been outlined in detail in the literature (Setlow 2019).

### Spores are not killed but only slow to germinate

Another problem that can cause incorrect conclusions about spore killing experiments is that while the treatment used appears to confer spore killing, the spores are not actually dead but only incapable of (or are very slow in) germination, as noted above for spores treated with strong alkali (Setlow et al. 2002). This is of great concern in applied settings, as very slow germination of what are thought to be completely dead spores can lead to severe consequences. Thus, with any new spore killing regimen it is crucial to be sure that the killed spores: (i) can still germinate; and (ii) if they cannot germinate in response to normal treatments, use artificial germinants such as CaDPA or low levels of lysozyme on recovery plates to be sure the spores are not “conditionally dead”. If these latter additions do germinate the “killed” spores and they are still inviable, then the spores’ death is not due to a failure to germinate. In this regard, note that as was described above, dead spores killed by a variety of agents including radiation and wet or dry heat, and even some chemicals, do still germinate, albeit never proceeding subsequently through outgrowth (Coleman et al. 2007, 2010; He et al. 2018; Setlow et al. 2013, 2016). The risk associated with such spores from a food safety perspective is very low, although the presence of high numbers of spores, even if dead, should be viewed with caution since this may reflect poor practice at some point in the process. The presence of pre-formed toxins associated with certain pathogenic species, and which are often produced around the time of sporulation, also has to be considered.

### The killing treatment triggers spore germination

A third concern that has bedeviled several studies of spore killing is that it is possible that the treatment used somehow triggers spore germination, and it is the more sensitive germinated spores that are then killed. This flaw was uncovered in analysis of how black silicon nanopillars kill spores, as it was only germinated spores that were being killed and these were formed during the treatment, while truly dormant spores were completely resistant (Ghosh et al. 2017). Germination followed by killing is also associated with some disinfectants, for example the cationic surfactant cetyltrimethylammonium bromide (CTAB) (Dong et al. 2019), albeit to a lesser extent in this example than observed for black silicon nanopillars. Simple phase contrast microscopy of spores before and after treatment will guard against this type of error, as dormant spores appear phase bright, while germinated spores are phase dark.

### Failure to remove or neutralize the potential killing agent

It is simple to immediately halt spore killing with radiation or heat by turning off the radiation source or rapid sample cooling. However, the situation with chemical agents can be more complicated, as the agent can adsorb to dormant spores and then kill the spores when they are exposed to germinants and nutrients to allow measurements of spore killing. Indeed, there have been agents suggested to kill dormant spores, but actually do not, as when the agent was either neutralized or well removed from the treated dormant spores, they were fully alive; one example being illustrated in Green et al. (2020). Thus, in analysis of any chemical treatment of spores, it is essential that the chemical be either neutralized or removed prior to spores’ conversion to the much less resistant germinated spores.

### Ascribing visible damage to spores as the cause of spore killing

Another common flaw in spore killing research can show up when microscopy is used to assess damage to spores, with this often being electron microscopy. In particular, some studies have seen physical damage to spores during treatment, one example being gas plasma which can cause morphological damage to spores, but this occurs only well after spore death (Yardimci and Setlow 2010). While spore killing by physical damage is certainly a possibility, this requires a quantitative demonstration that the percentages of spores that are damaged are equal to the percentages of spores that are killed, and this is very rarely done. In addition, this type of evidence is really only a correlation, and proving causal relationships between the physical damage and spore killing requires studies of other spore properties. Indeed, hydrogen peroxide kills spores without generation of any obvious damage.

## Areas for further investigation

Work over the past years has given us much information on agents that can kill spores, as well as an understanding of how some agents kill spores and how spores resist killing. However, there remain a number of areas in which more knowledge is needed.

In the area of mechanisms of spore killing, DNA damage by some agents has been reasonably well studied, especially for use of UV at several wavelengths (see above). However, although it is clear that γ-radiation kills spores by DNA damage, there has been minimal work identifying the precise DNA damage generated, such as strand breaks and oxidized bases in particular on guanine residues. The precise mechanism of wet heat killing of spores is also not clear. The identification of the central metabolic enzyme(s) thought to be involved would represent an important achievement, as knowledge of which spore protein is the weak link in spore core heat resistance might suggest strategies to improve the efficacy of spore wet heat killing. Indeed, work ~ 40 years ago indicated that a number of central metabolic enzymes in *B. cereus* have rather different wet heat stability in spores of this organism, and this is also reflected in these enzymes’ heat resistance in spore extracts (Warth 1980). Inactivation of a key core protein is also thought to underpin aqueous hydrogen peroxide mediated killing of spores, although again this protein has not been identified. Spore component(s) damaged by hydrogen peroxide vapour have also yet to be identified.

Several chemical agents, mentioned above and detailed in Table 1, including commonly applied oxidizing agents such as hypochlorite and chlorine dioxide, most likely kill spores via damage to the IM. However, the precise nature of this damage has not been established. It is not, however, associated with oxidation of unsaturated fatty acids, as spores with no IM unsaturated fatty acids are as sensitive to oxidizing agents as are wild-type spores (Cortezzo et al. 2004). The only other obvious IM component that could be damaged by oxidizing agents is one or more spore proteins, but as with wet heat there is no indication of the IM protein damage to which might result in spore killing. Again, this is clearly a matter for further investigation.

Another area in which research progress is also needed is for the “germinate to eradicate” strategy. If the goal is to eradicate spores of only one species/strain with known germination characterization, then germinants for spores of that organism can be used to stimulate the generation of the much less resistant germinated spores which are relatively easy to kill. This strategy has been shown to work well with spores of *B. anthracis* using the GR-germinant L-alanine to trigger germination and in broad areas the germinated spores even die off without further treatment needed (Buhr et al. 2020). However, the presence of superdormant spores in populations that germinate extremely slowly may well restrict this type of treatment to only 2–3 logs of spore killing. This strategy has also been suggested for *C. difficile* spores using bile salts to stimulate germination of these spores making them sensitive to killing by disinfectants such as ethanol and chlorhexidine (Budi et al. 2021; Nerandzic and Donskey 2010, 2013; 2015; Shen 2020). It is, however, not known if *C. difficile* spore populations contain low levels of spores that are superdormant for bile salt germination. Another treatment that uses the “germinate to eradicate strategy” for spore killing is high hydrostatic pressure (HHP), generally at > 600 megaPascals, which triggers spore germination by opening the SpoVA channel for CaDPA in spores’ IM (Black et al. 2007; Doona et al. 2016; Li et al. 2019; Modugno et al. 2019; Morimatsu et al. 2019; Paidhungat et al. 2002). Since CaDPA release from the spore core results in a significant decrease in spore wet heat resistance, if HHP treatment is carried out at elevated temperatures this results in spore killing, presumably by inactivating the CaDPA-less spores by damaging one or more essential spore core proteins, although this latter point has by no means been shown. Notably HHP is used to produce shelf-stable foods, and if carried out at high temperatures holds the potential for generating sterile foods.

As noted above, one concern about the general germinate to eradicate strategy is the presence of low levels of superdormant spores in spore populations that germinate only very slowly to physiological germinants. One approach to evade this concern is to use a “nonphysiological” germinant that germinates spores of all species. One agent that does this is dodecylamine and this agent has also been shown recently to also kill spores, and by a mechanism different than spore germination (Mokashi et al. 2020). However, whether such an agent would work in applied settings, in particular broad area decontamination, is not clear.

A final area of concern going forward is the appearance of new strains that make spores with much greater resistance properties. Indeed, this is the case for spores of some *Bacillus* strains that can resist normal food processing temperatures used for wet heat (Krawczyk et al. 2016, 2017; Li et al. 2019; Tu et al. 2021). It seems even likely that food processing regimens have selected for the highly resistant spores. Do these highly resistant spores have an Achilles heel that may allow them to be killed rapidly and easily by some other agents? What is/are the mechanisms associated with the greatly increased resistance of these spores? These are research questions that should be addressed going forward.

In addition to all the conclusions and points raised above, there are a number of additional methods of spore killing, that while not yet in routine use, may have promise for the future (Cho and Chung 2020; Reineke and Mathys 2020). Agents that fall into this group include enzymes (Fu et al. 2020; Mundra et al. 2014), various types of plasma (Kramer et al. 2020; Takamatsu et al. 2015; Wang et al. 2016), supercritical gasses (usually carbon dioxide) (Setlow et al. 2016), thermosonication (Fan et al. 2019), and ohmic heating (Schottroff et al. 2019). Notably the latter was recently shown to be more effective than heat alone at equivalent temperatures. Studies of the efficacy and mechanisms of these newer treatments are certainly warranted, in particular on how these treatments can be carried out in applied settings.

## Conclusion

Problems associated with spores, and more pertinently, how to kill them in a sector-appropriate manner, evidently remain. Encouragingly, the present review of the literature indicates that the search for solutions to spore-associated problems, and the accumulation of fundamental knowledge to assist in this quest, remains a healthy and vibrant area of research. The challenge moving forward will be to ensure that insight gained from rigorous lab-based research is rapidly translated to improved and preferably environmentally considerate spore inactivation procedures, meaning greater engagement between spore biologists and relevant stakeholders.
